# Renal Preservation Therapy for Renal Cell Carcinoma

**DOI:** 10.1155/2012/123596

**Published:** 2012-08-23

**Authors:** Yichun Chiu, Allen W. Chiu

**Affiliations:** ^1^Division of Urology, Department of Surgery, Taipei City Hospital, Zhongxiao Branch, Taipei 115, Taiwan; ^2^School of Medicine, National Yang-Ming University, Taipei 112, Taiwan

## Abstract

Renal preservation therapy has been a promising concept for the treatment of localized renal cell carcinoma (RCC) for 20 years. Nowadays partial nephrectomy (PN) is well accepted to treat the localized RCC and the oncological control is proved to be the same as the radical nephrectomy (RN). Under the result of well oncological control, minimal invasive method gains more popularity than the open PN, like laparoscopic partial nephrectomy (LPN) and robot assisted laparoscopic partial nephrectomy (RPN). On the other hand, thermoablative therapy and cryoablation also play an important role in the renal preservation therapy to improve the patient procedural tolerance. Novel modalities, but limited to small number of patients, include high-intensity ultrasound (HIFU), radiosurgery, microwave therapy (MWT), laser interstitial thermal therapy (LITT), and pulsed cavitational ultrasound (PCU). Although initial results are encouraging, their real clinical roles are still under evaluation. On the other hand, active surveillance (AS) has also been advocated by some for patients who are unfit for surgery. It is reasonable to choose the best therapeutic method among varieties of treatment modalities according to patients' age, physical status, and financial aid to maximize the treatment effect among cancer control, patient morbidity, and preservation of renal function.

## 1. Introduction

With the improvement of the detection modalities (ultrasound, high-quality computed tomography, etc.), the cases of small renal mass (SRMs) increased. In imaging study, 20% highly suspected renal malignancy would be finally proved as benign pathology after operation. On the other hand, studies proved that the more remaining kidney tissue we have, the lower prevalence the chronic kidney disease (CKD) would happen. Thus treatment gradually focused on the renal preservation therapy. To treat the patients with SRMs, three factors should be balanced: patient morbidity, preservation of renal function, and cancer control. In surgical part, nephron-sparing surgery (NSS)/partial nephrectomy (PN) have evolved from the treatment option to the standard management for small renal masses, have been shown to have equivalent oncological efficacy as radical nephrectomy (RN), while reducing the prevalence of the subsequent renal insufficiency. With the same oncological control, the goal of current surgical intervention is to decrease the risk of CKD. Preservation of renal function may be associated with improved survival and avoided of the risk of cardiovascular death. In ablative therapy, radiofrequency ablation (RFA), and cryoablation (CA) remained the role for high-risk group to reduce interventional morbidity. Active surveillance also play an role for the elderly, or high-risk patients, who are unfit for further intervention.

## 2. What Is the Role of Active Surveillance (AS)?

Urologists usually ask why AS for small renal tumors. The incidence of renal cancer increased, and they caused the medical fees expansion. Despite increasing surgeries were performed, the mortality rate for all renal tumors still increased [1]. Two questions must be taken into consideration now: are we treating more SRMs without impacting on mortality? Are we overtreating the harmless renal tumors? Hollingsworth et al. had analyzed the competing-cause mortality after the surgery of kidney cancer, they found 5% cancer-specific death (CSD) within 5 years in renal cancer smaller than 4 cm and 18% CSD in the group with size larger than 4 cm. Despite surgical therapy, competing-cause mortality for patients with renal masses rises with patients' increasing age. After 5 years, 28% elderly patients (> or =70 years) will die from other causes, suggesting the need for prospective studies to evaluate the role of active surveillance as an initial therapeutic approach for some SRMs [2]. What is the nature of SRMs? Only 10–30% renal masses regardless of the size are benign, but tumors in the elderly and SRMs are more likely to be benign [3]. Imaging modalities especially multidetector computed tomography (MDCT) scan revealed >90% accuracy of detection for RCC. But it is still controversial to distinguish between RCC and benign in growth or tumor size at presentation. 10% of SRMs <3 cm are pT3 and aggressiveness increased significantly when the renal tumor beyond 3 cm, but most SRMs are pT1 [4]. Renal biopsy is currently proved safe and low risk of tumor seeding even with the method of core biopsy. 92% biopsy result was conclusive. Thus renal tumor biopsy is suggested if the result may change the treatment policy such as lymphoma, metastasis of other cancer, or benign lesion [5]. 

Age and Charlson score (not treatment type) impacted overall survival on the study with multivariate analysis, and cardiovascular events were usually a leading cause of mortality, driven by chronic renal failure (CRF) [15]. It should be considered if the patient will have the probability to die of the other unrelated disease. Baseline clinical characteristics of SRMs do not allow to reliably predict the clinical outcome but available AS studies showed the chance of progression and metastasis is low in these selected cases (0.9%–1.3%). CT and MRI are thought to be more accurate in determining size variations and expert opinion advises 6~12 months interval as the optimal compromise between oncological safety and the risk of radiation exposure and kidney function morbidity due to contrast medium. Usually significant proportion of SRMs (30%) do not grow at all and the average grow is slow (0.15–0.3 cm/year), but if any growth up to 4 cm and doubling time of SRM volume <12 months are considered to terminate AS. Current SRM studies under AS disclosed small risk of progression (1-2%), who progress “tend” to grow faster and almost all death are noncancer related [16–20]. No clinically relevant tumor marker could help the AS now. Patients should know the following issues: SRMs are most malignant and RCC is life threatening. The aggressiveness cannot be precisely predicted. Growth rate does not predict malignancy and some SRM will show fast growth and aggressive behavior during surveillance. They also should know possible window of opportunity for NSS may be lost, and loss the opportunity of cure if metastases occur.

Active surveillance is a reasonable option for patients with limited life expectancy or for those who are unfit for or do not desire intervention. In the future, it will be helpful to know more about the natural history of untreated small renal masses. Developing inclusion and termination criteria is important to decrease the calculated risk of the cancer progression.

## 3. Ablative Therapy in Renal Preservation Therapy: Now and the Future? [Table 1]

Though the partial nephrectomy is the standard treatment for SRMs, the overall complication rate is around 20%. Thus less invasive percutaneous or laparoscopic ablation therapy such as cryoablation (CA) and radiofrequency ablation (RFA) are developed to be an alternative of surgical resection. The American Urological Association (AUA) guidelines regard the thermal ablation as the choice for the surgical high-risk patients who still wants to receive active treatment and accepts the need for long-term radiographic surveillance [21]. European Association of Urology (EAU) guidelines consider this modalities for the patients with SRMs and/or significant comorbidity who are unfit for surgery [22]. In these two famous guidelines, we could conclude that in patients with SRMs smaller 4 cm who are not suitable for PN (the elder or high comorbidity) or some cases with hereditary RCC (VHL syndrome), the ablative therapy is indicated.

Both CA and RFA could be done by laparoscopic or percutaneous (Perc) method. CA causes irreversible cell death at −70°C and RFA makes cell death and tissue coagulation with heating over 60°C The ideal mass to ablate is solid/enhancing and peripheral/exophytic mass, and the challenging cases were endophytic, hilar, and upper pole mass. Renal biopsy should be taken irrespective of technique and technology and choice of technique should be based on the situation and morphology of renal mass and patient comorbidity. The ideal case for laparoscopic approach is tumor in the anterior valve, upper pole and medial tumor nearby the ureter; good candidate for percutaneous ablation is the tumor in posterior valve, endophytic central and hilar area. The trend is to perform percutaneous method if you could do both of them.

In the current studies, cryoablation may have similar efficacy and complication rate as RFA [23]. The surgical (mainly laparoscopic, LCA) approach allows placement of cryotherapy probes into the lesions under direct vision. Real-time visual and continuous ultra sonographic monitoring provides precise control of the ice ball formation and extension. LCA is better to approach anterior and medical lesions to avoid of nearby tissue injury, but patients still need to receive general anesthesia (GA) and inherent risks of surgical exploration and dissection. Percutaneous CA (PCA) is ideal for posterior lesion and usually could be performed under sedation/local anaesthesia [24]. Comparing with PN, LCA results in higher risk of local tumor progression but a lower risk of perioperative complication [14]. No significant differences in efficacy were found between these two ablative techniques (RFA and CA) [23]. A problem arise from the ablation therapy is that correct pathology diagnosis could be missed due to the limited obtained tissue. Policy for followup after ablation is important no matter what kind of imaging modalities with or without biopsy. In cross sectional imaging, we need confirm absence of enhancement; CA lesion often shrinks with time but RF lesion may not shrink. Follow-up imaging studies are suggested in 3–6 and 12 months. Biopsy is suggested in case with suspicion of recurrence. In report from seven USA medical centers, the recurrence rate after RFA is 13.4% and CA is 3.9%. 2-year overall survival rate is 82.5% and disease-free survival is 97.4% [25]. Selection bias should be overcome for the future study comparing ablation therapy to PN. Ablation is accepted as therapy for SRMs with both short- and intermediate-term oncological efficacy [26], and long-term data is promising but limited [7, 8, 10–12]. Overall major complication rate 0.8–4% (higher in Perc RFA) and minor complication rate 0.8–4% (higher in LCA) were reported [27]. Surgical salvage after failed thermal ablation have be proved challenging and could result in radical nephrectomy. In conclusion, thermoablative therapy and cryoablation are reasonable options for patients who are unfit for surgery with small renal tumors (<4 cm) and they play an important role in the renal preservation therapy to improve the patient procedural tolerance. Technique selection should be based on anatomical tumor characteristics. Followup consists on regular cross-sectional imaging as success is currently a proxy for “absence of enhancement.” major complication rate is acceptable and do not differ between techniques and technologies. Long-term oncological results are limited and no difference for LCA, LRF, and PRF. Because most ablative therapy is used on the patients with elder age and high risk, some patients will possibly die before the recurrence happens and loss of followup. It is challenging to compare the results between ablative therapy and partial nephrectomy. Table 1 summarized the mid to long term results of CA and RF. And Olweny et al. compared the results between RF and PN [14].

Novel modalities but limited to small number of patients include high-intensity ultrasound (HIFU), radiosurgery, microwave therapy (MWT), laser interstitial thermal therapy (LITT), and pulsed cavitational ultrasound (PCU). They are still under evaluation [21, 33–36].

## 4. What Is the Role of Partial Nephrectomy: Past, Now, and the Future? [Table 2]

In recent years, the management of localized kidney tumors has changed, resulting in a shift away from radical nephrectomy (RN) toward a more frequent use of partial nephrectomy (PN). Open partial nephrectomy (OPN) has already become the standard of care in the treatment of renal masses smaller than 4 cm as well as select tumors up to 7 cm. The 5-year cancer-specific survival rate of partial nephrectomy had been proved more than 95%, almost the same as radical nephrectomy [37]. In additional to providing equivalent oncological control, the preservation of healthy kidney increased the overall survival rate and decreased comorbid disease. But bleeding, positive surgical margin (PSM) still raised some discussion. In 1993, laparoscopic partial nephrectomy (LPN) was first reported by Winfield et al. [38] and McDougall et al. [39]. The criticize at then is its challenging technique and long learning curve. But with the improved laparoscopic surgical instrument, this minimal invasive approach could also achieve equivalent outcome with OPN and lower morbidity profile (less operative time, decreased blood loss, and a shorter hospital stay) [28]. Gill et al. found an overall complication rate of 18.6% for LPN across all patients, with a complication decreasing from 22.1% for initial procedures to 8.5% for the procedures after the learning curve had been reached. They performed 800 LPN and concluded as the following: results will improve with experiences, warm ischemia time (WIT) will reduce down 14.4 minutes (initially 31 minutes), but with the earlier removal of the hilar clamp will increased estimated blood loss (EBL) and blood transfusion [29]. But not all the urologist could do 800 laparoscopic partial nephrectomies in all his life. Every minute counts when the renal hilum is clamped during partial nephrectomy [40]. Ordinary WIT is suggested below 30 minutes but recent recommendation is less than 20 minutes [41]. “Zero ischemia” and “selective-clamping” technique were introduced to decrease the effect of WIT but both of them are technically dependent [42, 43]. In 2004, Gettman et al. first described robot assisted laparoscopic partial nephrectomy (RPN) [44]. RPN had foreshortened the learning curve than LPN. It was a big barrier from OPN to LPN because of the warm ischemia time (WIT) and the positive surgical margin (PSM). With the improvement of laparoscopic surgical modalities, intraoperative ultrasound, tissue glue/sealant, improvement of stitches material (barbed suture), high-coagulation energy, sliding-clip renorrhaphy, robot for reconstruction, and minimally invasive approaches for PN have been rapidly gaining popularity. RPN makes intracorporeal suturing easier even for subspecialized surgeons. Contemporary series show RPN provides equal functional and oncological outcome [30, 31]. In conclusion, partial nephrectomy is the mainstream treatment in the surgical part for renal preservation therapy, current trend is from open PN to minimal invasive PN with equal oncological control and less surgical morbidity. Ischemia time should be limited less than 20 minutes, but LPN is technique challenging and reserved only to experienced centers. RPN shortens the learning for the laparoscopic approach method; the midterm result disclosed the equal functional and oncological control [45]. But the cost is the core problem of RPN. It is no doubt that high technology leads the trend of partial nephrectomy, preferably with laparoscopic approach in experienced hands.

## 5. Conclusion

Surgery is still the gold standard in the management of SRMs. Nephron sparing surgery (NSS)/partial nephrectomy (PN) is the best option whenever it could be used. RFA/CA are reasonable options for those old and high-risk patients that till wish active treatment for SRMs. New modality like HIFU is still under evaluation. Active surveillance (AS) should be restricted to the patients with high-surgical risk, elderly, infirm or who refuses surgery. For NSS/PN, evolution is from open partial nephrectomy (OPN) to minimal invasive treatment (LPN, RPN). It is reasonable to choose the best therapeutic method among varieties of treatment modalities according to patients' age, physical status, and financial aid to maximize the treatment effect among cancer control, patient morbidity, and preservation of renal function.

## Figures and Tables

**Table 1:** Mid- to long-term results of cryoablation (CA) and radiofrequency (RF), and one group compared the results between radiofrequency and partial nephrectomy (PN).

	Method	Approach	RCC numbers	Mean followup (months)	Local recurrence	Relapse free survival	Cancer specific survival
McDougal et al. [6]	RF	P	20	55	1	94%	100%
Levinson et al. [7]	RF	P	18	62	3	81%	100%
Tracy et al. [8]	RF	P, L, O	53	53	5	91%	99%
Davol et al. [9]	CA	L, O	34	64	5	84%	100%
Weld et al. [10]	CA	L	22	46	0	100%	100%
Aron et al. [11]	CA	L	55	95	7	87%	89%
Guazzoni et al. [12]	CA	L	44	61	0	100%	100%
Beemster et al. [13]	CA	L	51	30	3	94.4%	100%
Olweny et al. [14]	RF	P, L	37	78	3	91.7%	97.2%
	PN	L, O	37	73	3	94.6%	100%

RCC: renal cell carcinoma; RF: radiofrequency; CA: cryoablation; P: percutaneous; L: laparoscopy; O: open; PN: partial nephrectomy.

**Table 2:** Main series of minimal invasive partial nephrectomy (laparoscopic or/and robot assisted laparoscopic method).

Method	Gill et al. [28, 29]			Benway et al. [30]	Dulabon et al. [31]		Long et al. [32]	
	LPN			RPN	RPN		LPN	RPN
Number	276	289	235	183	51	405	182	199
	99~03	04~06	07~08		Hilar	nonhilar	Complex tumors (RENAL score >7)	
Size (cm)	3.0	2.9	3.3	2.87	3.2	2.6	4.0	3.8
OP Time (minutes)	202	235	250	210	195	187	241	197
EBL (mL)	242	245	373	132	262	208	325	280
WIT (minutes)	31.9	31.6	14.1	23.9	26.3	19.6	23.2	22.4
Complication	23.9%	14.9%	10.6%	8.2%	2.4%	5.2%	4.9%	5.5%
Transfusion	14.1%	8.7%	15.3%	1%	2.4%	4.2%	14.3%	12.1%

LPN: laparoscopic partial nephrectomy; RPN: robot assisted laparoscopic partial nephrectomy; OP time: operative time; EBL: estimated blood loss; WIT: warm ischemia time; RENAL nephrometry score: to quantify the anatomical characteristics of renal masses on computerized tomography or magnetic resonance imaging.
